# Identification of CNS Injury-Related microRNAs as Novel Toll-Like Receptor 7/8 Signaling Activators by Small RNA Sequencing

**DOI:** 10.3390/cells9010186

**Published:** 2020-01-11

**Authors:** Thomas Wallach, Max Wetzel, Paul Dembny, Ori Staszewski, Christina Krüger, Alice Buonfiglioli, Marco Prinz, Seija Lehnardt

**Affiliations:** 1 Institute of Cell Biology and Neurobiology, Charité—Universitätsmedizin Berlin, Corporate Member of Freie Universität Berlin, Humboldt-Universität zu Berlin and Berlin Institute of Health, 10117 Berlin, Germany; thomas.wallach@charite.de (T.W.); max.wetzel@charite.de (M.W.); paul.dembny@charite.de (P.D.); christina.krueger@charite.de (C.K.); alice.buonfiglioli@charite.de (A.B.); 2 Institute of Neuropathology, Faculty of Medicine, University of Freiburg, 79106 Freiburg, Germany; ori.staszewski@uniklinik-freiburg.de (O.S.); marco.prinz@uniklinik-freiburg.de (M.P.); 3 Berta-Ottenstein-Programme for Clinician Scientists, Faculty of Medicine, University of Freiburg, 79106 Freiburg, Germany; 4 Signalling Research Centres BIOSS and CIBSS, University of Freiburg, 79106 Freiburg, Germany; 5 Center for Basics in NeuroModulation (NeuroModulBasics), Faculty of Medicine, University of Freiburg, 79106 Freiburg, Germany; 6 Department of Neurology, Charité—Universitätsmedizin Berlin, Corporate Member of Freie Universität Berlin, Humboldt-Universität zu Berlin and Berlin Institute of Health, 10117 Berlin, Germany

**Keywords:** microRNA, toll-like receptor, microglia, neurons, neurodegeneration, small RNA sequencing

## Abstract

Toll-like receptors (TLRs) belong to pattern recognition receptors, which respond to danger signals such as pathogen-associated molecular patterns or damage-associated molecular patterns. Upon TLR activation in microglia, the major immune cells in the brain, distinct signaling cascades trigger the production of inflammatory molecules, being a critical feature in neuroinflammation and neurodegenerative processes. Recently, individual microRNAs (miRNAs) were shown to act as endogenous TLR ligands. Here, we conducted systematic screening for miRNAs as potential TLR7/8 ligands by small RNA sequencing of apoptotic neurons and their corresponding supernatants. Several miRNA species were identified in both supernatants and injured neurons, and 83.3% of the media-enriched miRNAs activated murine and/or human TLR7/8 expressed in HEK293-derived TLR reporter cells. Among the detected extracellular miRNAs, distinct miRNAs such as miR-340-3p and miR-132-5p induced cytokine and chemokine release from microglia and triggered neurotoxicity in vitro. Taken together, our systematic study establishes miRNAs released from injured neurons as new TLR7/8 activators, which contribute to inflammatory and neurodegenerative responses in the central nervous system (CNS).

## 1. Introduction

Toll-like receptors (TLRs) belong to a family of pattern recognition receptors, which recognize pathogen-associated molecules, such as bacterial and viral components, and damage-associated molecules derived from necrotic cells and tumor tissue. TLR signaling pathways are crucial for the crosstalk between non-immune and immune cells in the central nervous system (CNS) and periphery [1,2]. Two different classes of TLRs have been identified: the ones located at the cell surface, and TLRs such as TLR7 and TLR8 located in endosomes, mediating an inflammatory response against invading viruses and bacteria by sensing GU- and AU-rich single-stranded RNA (ssRNA) [3,4]. In general, TLRs contain a ligand-sensing ectodomain composed of leucine-rich repeats (LRRs). They are anchored by a transmembrane domain, which is linked to a cytoplasmic Toll/IL-1 receptor (TIR) domain that allows for binding of specific adaptor proteins, thereby activating downstream cascades. Consequently, cytokines and chemokines are released from immune cells [3]. The canonical TLR pathway is regulated by (i) ligand binding to specific residues within the LRRs of TLR homodimers, (ii) conformational changes causing TLR dimerization, and (iii) consequent binding of adaptor proteins to cytosolic TLR parts. Subsequently, kinases are activated, thereby directing the transcription of inflammatory molecules. TLR7 and TLR8 are nucleic acid-sensing receptors that can be activated by ssRNA derived from the human immunodeficiency virus-1 (HIV-1) or influenza genomic RNA [5,6,7]. In this context, the most potent motif activating TLR7/8 was narrowed down to a GUUGUGU sequence fragment exhibiting the highest degree of activation in immune cells [5]. Interestingly, such or slightly mutated small TLR7/8 recognition motifs are also present in host-derived ssRNA species, such as microRNAs (miRNAs). The highly conserved miRNA *let-7b*, abundantly expressed in the brain, contains the exact GUUGUGU motif [8].

miRNAs are 19–24 nucleotide noncoding RNAs that usually bind to 3’-untranslated regions (UTRs) of RNAs promoting their degradation. To date, about 5000 mammalian sequences have been classified as miRNA, with still increasing numbers due to the rapid progress in sequencing-based approaches. Furthermore, levels of miRNA-mediated regulation reach an immense complexity as a single miRNA can target different RNA molecules, and a single 3’UTR can be occupied by different miRNA species [9]. We demonstrated in previous work that *let-7b* does not only function at post-transcriptional level, but also can serve as a signaling molecule in the CNS. This miRNA directly activates TLR7 in microglia, the major immune cell in the brain, and neurons. These interactions result in microglial TNF release, neuroinflammation, and neurodegeneration [8]. Additionally, copy levels of select members of the *let-7* miRNA family are elevated in the cerebrospinal fluid (CSF) of Alzheimer’s disease (AD) patients compared to control individuals, confirming the extracellular existence of these miRNAs in the setting of human neurodegenerative diseases, such as AD [10]. However, apart from *let-7b* able to activate TLR signaling in CNS cells [8] and detected in human CSF [10,11], the identity of further miRNAs acting as TLR signaling molecules in the context of CNS damage, remains unresolved.

In this study, we conducted a systematic approach to identify miRNAs as TLR7/8 signaling activators in the setting of CNS injury employing small RNA sequencing. Induction of apoptosis in murine cortical neurons serving as a proxy for neurodegeneration resulted in the release of miRNAs into the extracellular space. Subsequent sequencing analysis revealed 22 miRNAs in the supernatants derived from apoptotic neurons significantly altered compared to control, and eight miRNAs in the injured neuron samples whose expression was negatively altered compared to control. In a second step, 12 miRNAs enriched in the media of apoptotic neurons were tested for their ability to function as signaling molecules in murine or human TLR7/8-overexpressing HEK293-derived TLR reporter cells. Ten out of these 12 miRNA candidates (83.33%) activated murine TLR7 and/or human TLR8. Further, select miRNAs out of this miRNA pool activating murine/human TLR7 and human TLR8 induced the release of various cytokines and chemokines from murine microglia as well as TNF from human-derived monocytes. When extracellularly delivered in co-cultures of neurons and microglia, these miRNAs caused neuronal injury. Altogether, we identified distinct miRNAs as novel TLR7/8 activators involved in CNS injury, thereby providing mechanistic insight for a potential role of these miRNAs as signaling molecules in CNS diseases.

## 2. Materials and Methods

### 2.1. Mice and Ethics Statement

C57BL/6 mice were obtained from the FEM, Charité – Universitätsmedizin Berlin, Germany, and were used for the generation of primary microglial cultures, primary neuronal cultures, and co-cultures of neurons and microglia. TLR7 knocked out (KO) mice were generously provided by S. Akira (Osaka University, Japan). Animals were maintained according to the guidelines of the committee for animal care. All animal procedures were approved by the *Landesamt für Gesundheit und Soziales* (*LaGeSo*) Berlin, Germany.

### 2.2. Synthetic microRNA Mimics

RNA oligoribonucleotides were modified with 5’ phosphorylation and phosphorothioate bond in every base and purchased from IDT (Integrated DNA Technologies, Coralville, IA, USA). Sequence information is provided in Table 1 and Table S1. Sequences of control mutant oligonucleotides are given in brackets (UGAGGUAGAAGGAUAUAAGGAU or AAUCUCUACCCCAAGACCAAAA).

### 2.3. Primary Cortical Neurons

Cultures of purified cortical neurons were generated as described previously [8]. Briefly, neurons were isolated from the mouse cortex at embryonic day (E) 17.5. Brains were separated from blood vessels, meninges, and cerebellum and transferred into a 15 mL Falcon tube filled with HBSS buffer (Gibco #14170-088, Thermo Fisher Scientific, Waltham, MA, USA). After two washing steps, 500 µL of Trypsin (2.5%) was added for 20 min at 37 °C. Trypsin reaction was stopped with 4 mL of heat-inactivated fetal calf serum (FCS; Gibco #10082-147, Thermo Fisher Scientific, Waltham, MA, USA), cells were washed twice with HBSS and incubated with 100 µL DNase (1 mg/mL) for 1 min. Subsequently, cells were washed twice with Neurobasal Medium (Gibco #21103-049, Thermo Fisher Scientific, Waltham, MA, USA) supplemented with 1% L-Glutamin (Gibco #25030-024, Thermo Fisher Scientific, Waltham, MA, USA), 1% penicillin/streptomycin (Gibco #15140-122, Thermo Fisher Scientific, Waltham, MA, USA), and 2% B27 supplement (Gibco #17504-044, Thermo Fisher Scientific, Waltham, MA, USA). Neurons were then transferred into a new 50 mL Falcon tube, resuspended in 10 mL Neurobasal Medium and centrifuged at 300 rpm at 4 °C for 5 min. Supernatants were separated carefully from the pellet and transferred into a 50 mL Falcon tube. Neurons were placed in Neurobasal Medium and seeded into 6-well plates. On the next day, half of the media was replaced by new media, and cells were incubated for additional 48 h before the start of the experiment.

### 2.4. Primary Microglia

Microglia were dissected from brains of newborn mice (postnatal day (P) 0–4). Brain tissue was separated from blood vessels, meninges, and the cerebellum, and collected in a 15 mL Falcon tube (BD Falcon #352096, Franklin Lakes, NJ, USA) filled with L-15 Leibowitz washing buffer (Gibco #114115-049, Thermo Fisher Scientific, Waltham, MA, USA). After removing the L-15 buffer, tissue was supplemented with 3 mL 2.5% Trypsin (Gibco #15090-046, Thermo Fisher Scientific, Waltham, MA, USA) for 25 min at 37 °C. The trypsin reaction was stopped with heat-inactivated fetal calf serum (FCS; Gibco #10082-147, Thermo Fisher Scientific, Waltham, MA, USA), and 100 µL DNase was added to the tubes (1 mg/mL; Roche #ROD 1284932, Basel, Switzerland) for 1 min. Subsequently, the suspension was centrifuged at 1200 rpm at 4 °C for 5 min. Supernatants were removed, and Falcons were filled with Dulbecco’s modified Eagle’s medium (DMEM Invitrogen #41965062, Carlsbad, CA, USA) supplemented with 10% FCS and 1% antibiotics penicillin and streptomycin. Cells were mechanically dissociated suspended and were passed through a 70 µm cell strainer in a 50 mL Falcon. Finally, microglia were cultured in a cell culture flask with 10 mL DMEM. After 24 h, medium was changed, and after a further 7 days 5 mL of DMEM were added. After 10–14 days, microglia were isolated by gentle shaking and were plated into a 96- or 24-well plate. Experiments were performed 24 h after plating.

### 2.5. Co-Cultures of Neurons and Microglia

For co-culture experiments 6 × 10^4^ microglial cells were added to 5 × 10^5^ neurons, which were seeded on a PDL-coated coverslip in a 24-well plate. On the next day, cells were stimulated with synthetic oligoribonucleotides as indicated in the figures using LyoVec (InvivoGen #LYEC-RNA, San Diego, CA, USA) as complexation reagent following the manufacturer’s protocol. After 3 d, cells were washed 3x with PBS and fixed with 4% PFA.

### 2.6. Immunofluorescence Microscopy

4% PFA-fixed cells were washed with PBS and incubated overnight at 4 °C with primary antibody against neuronal nuclei (NeuN; Millipore #MAB377, Burlington, MA, USA) in permeabilization solution (PBS, 2% normal goat serum, 0.2% TritonX-100). On the next day cells were washed 3x with PBS and incubated for 1 h with the secondary antibody. Subsequently, nuclei were stained with DAPI (4’, 6-diamidino-2-phenylindole) solution (1:10,000; Sigma Aldrich #D9542, St. Louis, MO, USA) for 1 min. Cells were mounted and analyzed by an epifluorescence microscope (Olympus BX51, Tokyo, Japan).

### 2.7. Induction of Apoptosis in Neurons

Cortical neurons cultured in Neurobasal Medium (Gibco #21103-049, Thermo Fisher Scientific, Waltham, MA, USA) were treated with 1 µM (final concentration) staurosporine (Merck #S6942, Kenilworth, NJ, USA) or solvent control (DMSO) for 8 h. Subsequently, media were separated from cells, and both neurons and their corresponding media were stored at −80 °C.

### 2.8. Small RNA Enrichment

Small RNA enrichment was performed as previously described [12]. Briefly, 700 µL of Trizol (Thermo Fisher Scientific #15596026, Waltham, MA, USA) was added to 1 mL neuron-conditioned media (supernatant of neurons) or neuron samples. Samples were vortexed for at least 10 s and allowed to rest at room temperature for an additional 5 min. Subsequently, 140 µL of chloroform was added, and samples were shaken for additional 15 s and centrifuged at 14,000 rpm at 4 °C for 15 min. The aqueous phase was transferred to a new tube without touching the interphase. Small RNA enrichment was achieved using the mirVana (Thermo Fisher Scientific #AM1560, Waltham, MA, USA) isolation kit according to the manufacturer’s protocol. RNA quantity and quality were determined using Nanodrop 2000c (Thermo Fisher Scientific, Waltham, MA, USA) and the Agilent 2100 Bioanalyzer system (Agilent Technologies, Inc., Santa Clara, CA, USA) with the Agilent 2100 Small RNA Chip Kit (#5067-1548).

### 2.9. Library Generation and Small RNA Sequencing

Library preparation and small-RNAseq were performed at the KFB - Center of Excellence for Fluorescent Bioanalytics, University of Regensburg, Regensburg, Germany; www.kfb-regensburg.de, and were carried out according to the TruSeq small RNA Library Preparation Kit Reference Guide (Illumina, Inc., San Diego, CA, USA), the Illumina HiSeq 1000 System User Guide (Illumina, Inc., San Diego, CA, USA), and the KAPA Library Quantification Kit - Illumina/ABI Prism User Guide (Kapa Biosystems, Inc., Woburn, MA, USA). In brief, 10 ng of enriched small RNA (mirVana Kit) was used for ligation of the RNA 3’ and the RNA 5’ adapters, followed by reverse transcription to create single-stranded cDNA. cDNA was then PCR-amplified for 12 cycles using a common and an index primer. Instead of a gel purification of the small RNA libraries, a magnetic bead clean up (Agencourt AMPure XP, Beckman Coulter, Brea, CA, USA) with a final volume of 10 µL elution buffer was performed. Purity and integrity were assessed on the Agilent 2100 Bioanalyzer with the High Sensitivity DNA Assay (Agilent, Palo Alto, CA, USA). Libraries were quantified using the KAPA SYBR FAST ABI Prism Library Quantification Kit (Kapa Biosystems, Inc., Woburn, MA, USA). Equimolar amounts of each library were pooled and were used for cluster generation on the cBot with the Illumina TruSeq SR Cluster Kit v3. The sequencing run was performed on an HiSeq 1000 instrument using the indexed, 50 cycles single-read (SR) protocol and the TruSeq SBS v3 Reagents according to the Illumina HiSeq 1000 System User Guide.

### 2.10. Data Processing

Adapter Trimming was performed on fastq files using trimmomatic 0.36 with standard commands (ILLUMINACLIP:TruSeq3-SE:2:30:10 LEADING:3 TRAILING:3 SLIDINGWINDOW:4:15 MINLEN:36). Trimmed Fastq files were aligned to the reference genome (Gencode M11) using STAR_2.5.2b. miRNA counts were generated using feature counts featureCounts v1.6.2 with the MirBASE 21 mouse dataset as differential miRNA expression using DESeq2 1.20. Raw data are available at NCBI GEO (GSE138532, https://www.ncbi.nlm.nih.gov/geo/info/linking.html).

### 2.11. HEK-Blue TLR Reporter Cell Lines

HEK-Blue TLR reporter and corresponding Null control cell lines were purchased from InvivoGen, San Diego, CA, USA. One day after seeding 50,000 cells/well in a 96-well plate, HEK-Blue SEAP reporter 293 cells expressing mTLR7, mTLR8, hTLR7 or hTLR8 and the inherent control cells were incubated with different miRNA mimics or control oligoribonucleotide (10 µg/mL) using LyoVec as tranfection agent (InvivoGen #LYEC-RNA, San Diego, CA, USA) according to the manufacturer’s protocol. Cells were stimulated with indicated agents dissolved in 90% HEK-Blue detection reagent and 10% culture media. Detection of the reporter protein secreted embryonic alkaline phosphatase (SEAP) was performed at 655 nm using Varioskan Flash (Thermo Fisher Scientific, Waltham, MA, USA). For each condition, four wells were individually measured, and the average was used for evaluation.

### 2.12. TNF Enzyme Linked Immunosorbent Assay

Microglia were isolated and plated into a 96-well plate at a density of 3 × 10^4^ cells/well. On the next day, microglia were incubated with synthetic mmu-miR-340-3p (5 µg/mL) or mmu-miR-132-5p (5 µg/mL) oligoribonucleotide complexed with LyoVec (InvivoGen #LYEC-RNA, San Diego, CA, USA) for various time intervals (9, 24, or 36 h) using various dosages (0.1, 1, 5, or 10 µg/mL). Subsequently, supernatants were collected and stored at −80 °C. TNF concentrations in the supernatants were assessed using the TNF mouse uncoated ELISA Kit provided by (Thermo Fisher Scientific, # 88-7324-88, Waltham, MA, USA) according to the manufacturer’s protocol.

### 2.13. Multiplex Immune Assay

To detect multiple cytokines and chemokines released from microglia in response to synthetic mmu-miR-340-3p and mmu-miR-132-5p after 24 h (concentration 5 µg/mL) the mouse custom ProcartaPlex 13-plex from (Thermo Fisher Scientific #PPX-15-MX2W762, Waltham, MA, USA) assay panel was used following the manufacturer’s protocol. The analysis included TNF, IL-6, IL-10, IL-1-β, GRO-α/KC, MIP-2, RANTES, GM-CSF, IP-10, MCP-1, MCP-3, IFN-α, and IFN-γ. Briefly, magnetic capture beads were washed, subjected to a 96-well plate and incubated with 50 µL of microglial culture supernatant. Samples were then centrifuged at 4 °C for 10 min at 1000× *g* and incubated with 50 µL assay buffer overnight. On the next day, after three washing steps detection antibody was added to the wells. Finally, beads were resolved in reading buffer, and detection was performed on a Luminex 200 device using the Bio-plex Software 4.0 (Bio-Rad, Hercules, CA, USA).

### 2.14. Gene Ontology and KEGG Analyses

GO slim categories and KEGG pathway enrichment for murine miRNAs (18 miRNA candidates from Table 1) were conducted using the DIANA-miRPath v3.0 software package (http://snf-515788.vm.okeanos.grnet.gr/). Significantly enriched GO terms and KEGG pathways were assessed using *p* < 0.05 as threshold [13].

### 2.15. Statistical Analyses

Significances of indicated groups compared to the corresponding control groups were determined by Student’s *t*-Test using GraphPad Prism 7.0 (GraphPad Software, San Diego, CA, USA).

## 3. Results

### 3.1. Identification of miRNAs Released from Apoptotic Neurons

**Figure 1:**
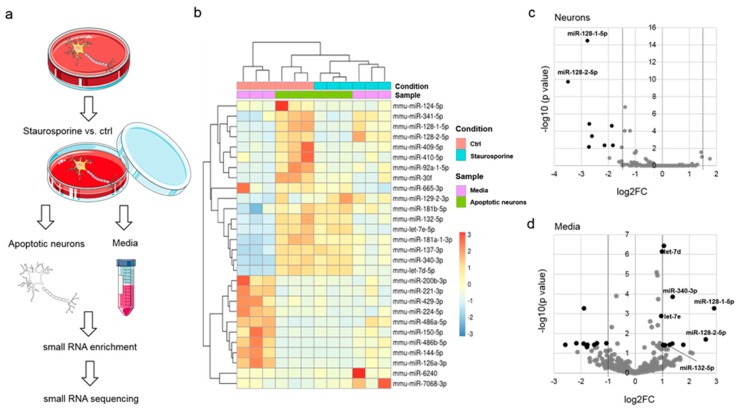
Analysis of miRNA release from injured cortical neurons into the extracellular space by small RNA sequencing. (**a**) Flow chart of apoptosis induction. Murine primary cortical neurons from C57BL/6 mice were incubated with either 1 µM staurosporine or DMSO (solvent control) for 8 h. miRNAs were isolated from the resulting supernatants (media) or apoptotic neurons followed by small RNA enrichment and small RNA sequencing (*n* = 3). (**b**) Heat map of significantly dysregulated miRNA species. Color scheme depicts Z-Score for each sample. (**c**) Volcano plot depicting significantly differentially present miRNAs in injured neurons (black dots: fold change FC > ±1.5; *p* < 0.05). (**d**) Volcano plot depicting significantly differentially present miRNAs in supernatants of apoptotic neurons (media; black dots: FC ≥ ±1.0; *p* < 0.05).

Dying neurons release distinct miRNAs, such as *let-7b* [8]. To identify further miRNAs that are able to activate nucleic-acid sensing TLRs in the CNS within a systematic approach, apoptosis was induced in murine primary cortical neurons by staurosporine, an established bacterial toxin causing programed cell death in neurons [14,15,16,17] (Figure 1a). To avoid an unspecific release of their whole nucleic acid content into the media at late stages of cell death, staurosporine- and control-treated neurons were analyzed after a limited period of 8 h. At this time point, according to visual inspection, neurons morphologically remained intact (Figure S1a). Next, small RNAs present in the neuronal supernatants and the small RNA fraction derived from the apoptotic neurons were isolated. For small RNA enrichment, a dual isolation strategy was pursued. First, total RNA was isolated through organic extraction followed by immobilization, and second, enrichment of small RNAs (<200 nucleotides) was achieved using glass fiber columns [12]. To ensure the quantity and quality of the isolated oligoribonucleotides several photometric approaches were used, as indicated (Figure S1b). After small RNA enrichment, Illuminia HiSeq smallRNA sequencing was performed (Table S1). Based on principal components analysis miRNAs identified by small RNA sequencing formed specific clusters (Figure S2a). Further, while no miRNAs were found enriched, eight miRNAs were significantly less present in apoptotic neurons, setting a cut-off at log2FC > ±1.5 and *p* < 0.05 (FC-fold change) (Figure 1b,c and Figure S2). Among these miRNAs, the two paralogues miR-128-1-5p and miR-128-2-5p showed the highest degree of negative expression alteration (Figure S2b). We detected 22 miRNAs that were differentially altered in the supernatants of apoptotic neurons compared to control conditions (log2FC ≥ ±1, *p* < 0.05) (Figure 1d and Figure S2). Focusing on the identification of miRNAs as signaling molecules for TLR7/8, we considered miRNAs enriched in the supernatants of apoptotic neurons as potential TLR ligand candidates. Among the identified 12 miRNAs significantly enriched in the media compared to control were *let-7d* and *let-7e*, two members of the *let-7* miRNA family, which have been previously reported as signaling molecules for TLR7 in microglia and macrophages (Figure 1d) [8,10]. In addition, miR-181a-1-3p and miR-181b-5p, two members of the miR-181 family, which have been previously observed to contribute to CNS inflammation at post-transcriptional level in astrocytes (Table 1) [18], were identified.

Virus-derived GU-rich nucleotide sequences were the first to be identified to activate human TLR7 and human TLR8 [5]. In addition, Forsbach and colleagues systematically determined GU-containing 4-mers to preferentially activate human TLR7/8, while AU-rich 4-mers seemed to be restricted to human TLR8 activation in immune cells [4]. Based on this, we determined the GU and AU content of the miRNA candidates identified by small RNA sequencing described above, as summarized in Table 1. Most of the miRNAs, except miR-6240, miR-129-2-3p, and mmu-mir-30f, exhibit a GU-content above 40% while the highest degree of GU-content, reaching 77.3%, is present in *let-7e-5p*. miR-137-3p contains the highest degree of AU content (65%), whereas the miR-128 family members contain A and U to a lesser extent (approximately 30%). Of note, several of the detected miRNAs identified in supernatants and neurons harbor specific motifs previously described to activate hTLR8 or hTLR7/8 (see Table 1) [4]. miR-340-3p harbors two, in part overlapping sequences (UUAU and UAUA), which are assumed to activate hTLR8, and an UCUC motif that was shown to activate hTLR7/8. In contrast, mmu-miR-132-5p contains two hTLR7/8 activating motifs (UUUC and UUGU), while in the case of mmu-miR-137-3p two sequences restricted to hTLR8 activation overlap (UUAUU).

To assess how the miRNAs identified above are embedded into the molecular regulatory context we performed KEGG and GO analysis using mirPath v.3 [13]. For enrichment analysis, we considered a list of 18 miRNAs including the ones that were either enriched in the supernatant or less present in neurons, both groups containing recognition motifs for hTLR7/8 (see Table 1). KEGG enrichment analysis revealed the involvement of these miRNAs in e.g., MAPK- and mTOR-signaling pathways that have been previously linked to TLR-mediated downstream processes [19,20] (Table S2). In addition, GO categories like cell-cell signaling and cytoplasmic membrane-bound vesicle and cell death point to a potential role of the identified miRNAs as signaling molecules (Table S2).

### 3.2. Select miRNAs Released from Injured Neurons Activate TLR7/8 Expressed in HEK293-Derived TLR Reporter Cells

**Figure 2:**
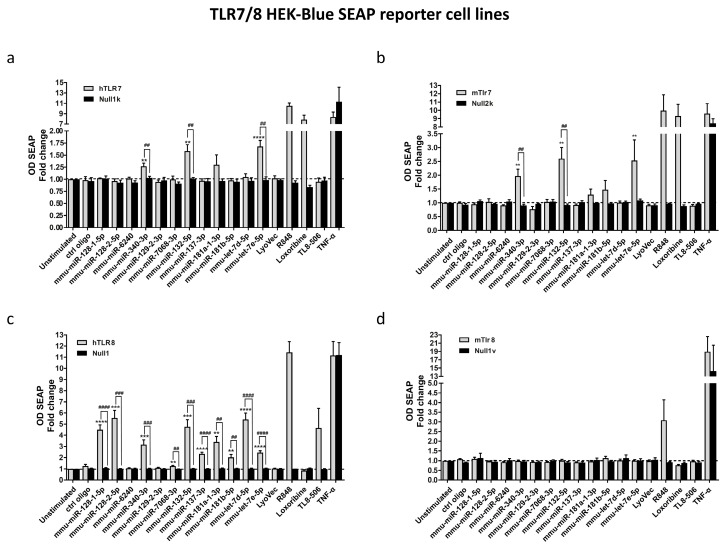
Extracellularly delivered miRNAs activate TLR7/8 in HEK293-derived TLR reporter cells. Human (**a**,**c**) or murine (**b**,**d**) TLR7 or TLR8 HEK-Blue-secreted embryonic alkaline phosphatase (SEAP) reporter cells and the corresponding null control cell lines were incubated with 10 µg/mL of the indicated synthetic oligoribonucleotides identified from small RNA sequencing analysis of supernatants from apoptotic neurons for 24 h. LyoVec and unstimulated cells served as negative controls, while loxoribine (TLR7), resiquimode (R848; TLR7/8) and TL8-506 (TLR8) served as positive control for the respective TLR reporter. Mutant oligoribonucleotide (ctrl oligo, 10 µg/mL) was used as a negative control. TNF was applied to activate the NF-κB/AP-1 promoter in reporter and control cells. SEAP protein was detected at an optical density (OD) of 655 nm and depicted as fold change of the SEAP protein normalized to unstimulated control. (**a**) Response of human TLR7 HEK-Blue reporter and control cells (Null1k) to indicated miRNAs. (**b**) Response of murine TLR7 HEK-Blue reporter and control cells (Null2k) to indicated miRNAs. (**c**) Response of human TLR8 HEK-Blue reporter and control cells (Null1) to indicated miRNAs. (**d**) Response of murine TLR8 HEK-Blue reporter and control cells (Null1v) to indicated miRNAs. Depicted are *n* = 4–6 individual experiments. Significances were obtained by Student’s *t*-Test. (* *p* < 0.05, ** *p* < 0.01, *** *p* < 0.001, **** *p* < 0.0001; * compared to unstimulated control; # compared to HEK Null parental control cells). Data are represented as mean ± s.e.m.

We hypothesized that miRNAs released from apoptotic neurons into the media and harboring GU-rich sequence motifs act as extracellular signaling molecules for TLR7/8. To test, whether the 12 miRNAs significantly enriched in the media compared to control, are able to serve as TLR7/8 signaling activators, we made use of HEK-Blue reporter cells overexpressing human TLR7, human TLR8, murine TLR7, or murine TLR8. In this reporter system, TLR7/8 activation is displayed by transcription of the *secreted* embryonic *alkaline phosphatase* (SEAP) caused by NF-κB/AP-1 activation, a well-described output of the canonical TLR signaling cascade [3]. The imidazoquinoline resiquimode (R848) [21], an established TLR7/8 activator, loxoribine, a selective agonist for TLR7 [22], and TL8-506, a selective TLR8 ligand [23], served as positive controls within our experimental set-up. A mutated oligoribonucleotide (ctrl oligo) with reduced GU content [8] was used as a negative control, whereas TNF was used as a control for NF-κB/AP-1 promoter activation. In contrast to the control mutant oligoribonucleotide, 10 out of the 12 tested miRNA candidates activated human/murine TLR7/8 (Figure 2c). In particular, miR-132-5p, miR-340-3p, and *let-7e* oligoribonucleotides induced significant activation of human TLR7, murine TLR7, and human TLR8, while the respective parental HEK Null control cells did not show such a response (Figure 2a–c). Of note, the TLR7/8-activating miRNAs miR-132-5p, miR-340-3p, and *let-7e* harbor at least two TLR7/8 recognition motifs previously described by Forsbach et al. [4] (Table 1). Interestingly, although not reaching significance, the two tested miR-181 family members also tended to activate human/murine TLR7 (Figure 2a,b). Furthermore, miR-128-1-5p and miR-128-2-5p, the most enriched miRNAs in the neuronal supernatants, being less present in neurons, and lacking established TLR7/8-activating sequences, induced profound activation of human TLR8 (Figure 2c). miR-137-3p, which contains TLR8-recognizing motifs (UUAUU), activated human TLR8 exclusively (Figure 2c). Murine TLR8 was not activated by any of the tested miRNA candidates (Figure 2d), as expected, and in line with previous reports on this receptor considering it nonfunctional [21,24].

In summary, approximately 80% of the miRNA candidates enriched in supernatants of apoptotic neurons and identified by small RNA sequencing activated TLR7 and/or TLR8, in a sequence-specific fashion.

### 3.3. miR-340-3p and miR-132-5p Induce Cytokine and Chemokine Release from Murine Microglia and Human-Derived Monocytes

**Figure 3:**
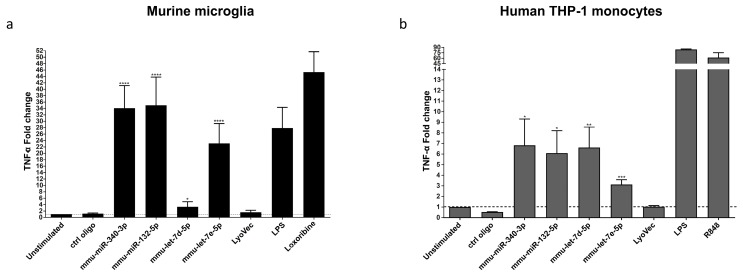
miR-340-3p and miR-132-5p induce TNF release from murine microglia and human THP-1 cell-derived monocytes. Murine microglia from C57BL/6 mice (**a**) and human THP-1-derived monocytes (**b**) were incubated with 10 µg/mL of the indicated miRNAs for 24 h. Loxoribine (TLR7), resiquimode (R848; TLR7/8), and lipopolysaccharide (LPS) (TLR4) served as positive controls, while unstimulated condition and LyoVec were used as a negative control. Mutant oligoribonucleotide (ctrl oligo, 10 µg/mL) was used as a further negative control. TNF amounts in supernatants were determined via ELISA and are shown as fold change normalized to unstimulated control. Significances were obtained by Student’s *t*-Test. (* *p* < 0.05, ** *p* < 0.01, *** *p* < 0.001, **** *p* < 0.0001; * compared to unstimulated control). Data of *n* = 3 are represented as mean + s.e.m.

To investigate the ability of the miRNAs identified as new TLR7/8 ligands to mediate an inflammatory response in the CNS in principle, murine microglia, which express all TLRs identified so far, including TLR7 [25], were incubated with synthetic miR-340-3p and miR-132-5p. These two miRNAs were chosen based on their potential to active both murine and human TLR7, as well as human TLR8 in our TLR reporter cell system (see above). *Let-7d* and *let-7e* previously shown to activate TLR7/8 in macrophages served as positive control for miRNA-induced TLR activation [8]. Additionally, lipopolysaccharide (LPS) as known activator of TLR4, served as a further positive control for microglial activation. After 24 h, microglial supernatants were analyzed for TNF by ELISA. miR-340-3p, miR-132-5p, and *let-7e* significantly induced TNF release from microglia, and these responses were similar to the one induced by loxoribine, the established TLR7 agonist (Figure 3a). Moreover, miR-340-3p- and miR-132-5p-induced TNF release occurred in a time- and dose-dependent fashion (Figure S3a–d). These effects required TLR7 expression, as TLR7-deficient microglia did not respond to miR-340-3p or miR-132-5p treatment (Figure S3e) [8].

To investigate whether microglia release further inflammatory molecules besides TNF in response to miR-340-3p and miR-132-5p, supernatants of microglia exposed to the respective miRNAs were analyzed by multiplex immune assay. Both miRNAs induced the release of TNF, Gro-α /KC, IL-6/10, IP-10, MCP-1/-3, MIP-2, and RANTES (Figure S4). To confirm the potential of miR-340-3p and miR-132-5p to activate human immune cells, we tested the response of human THP-1 cell-derived monocytes known to express TLR8 [26,27], to the miRNAs named above. Both miR-340-3p and miR-132-5p induced TNF release from these cells (Figure 3b). Altogether, miR-340-3p and miR-132-5p identified as new TLR7/8 signaling activators induced a specific cytokine and chemokine response in murine microglia and human monocytes.

### 3.4. miR-340-3p and miR-132-5p Trigger Neuronal Injury

**Figure 4:**
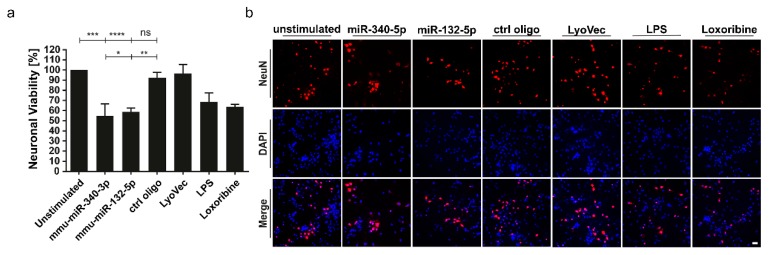
miR-340-3p and miR-132-5p trigger neuronal cell death in co-cultures of neurons and microglia. (**a**,**b**) Murine neuron/microglia co-cultures derived from C57BL/6 mice were incubated with 5 μg/mL of mmu-miR-340-3p, mmu-miR-132-5p, or control mutant oligoribonucleotide (ctrl oligo) for 3 days. LPS (100 ng/mL) and loxoribine (1 mM) were used as positive control, while LyoVec and unstimulated condition served as negative control. Subsequently, cells were stained with NeuN antibody and DAPI. (**a**) Relative neuronal viability was assessed by quantification of NeuN-positive cells. Significances were obtained by Student’s *t*-Test. * *p* < 0.05, ** *p* < 0.01, *** *p* < 0.001, **** *p* < 0.0001; * compared to unstimulated control. Data of *n* = 3 are represented as mean ± s.e.m. (**b**) Representative images of neuron/microglia co-cultures from C57BL/6 mice immunostained as described above. Scale bar, 20 µm.

We showed in previous work that *let-7b* serving as a TLR7 ligand induces neurodegeneration [8]. To test whether miR-340-3p and miR-132-5p identified as new signaling activators of TLR7/8 can mediate neuronal injury, co-cultures of neurons and microglia were treated with the miRNAs named above complexed with LyoVec. Mutated control oligoribonucleotide served as negative control, whereas loxoribine and LPS were used as established mediators of neuronal injury through TLR7 and TLR4 signaling, respectively [28,29]. Subsequently, cells were immunostained using an antibody against neuronal nuclei (NeuN), and stained with DAPI. Quantification of NeuN-positive cells revealed a significant loss of neurons in cell cultures exposed to miR-340-3p or miR-132-5p compared to control conditions (Figure 4a,b). These neurotoxic effects required the presence of microglia, as cell cultures of purified neurons did not show any reduction of neuronal numbers when exposed to miR-340-3p or miR-132-5p (Figure S5). These findings point to a functional role of miR-340-3p and miR-132-5p as signaling molecules in the setting of neurodegeneration.

## 4. Discussion

To date, only a few studies focused on miRNAs as potential signaling molecules in a systematic fashion, none of them addressing the role of miRNAs acting as TLR-activating signaling molecules in the CNS [30,31]. We identified in previous work an unconventional role for *let-7* miRNAs, which harbor GU-rich sequences, as direct activators of TLR7 [8]. Based on our previous findings that injured neurons release *let-7* that in turn can damage further neurons through TLR7 signaling [8], we hereby conducted a systematic approach to identify miRNAs as signaling molecules analyzing supernatants of apoptotic neurons for the presence of miRNAs by small RNA sequencing. In a second step, we tested all of the miRNAs found enriched in the supernatant of apoptotic neurons as potential TLR7/8 ligands. The confirmed miRNA candidates were then validated as signaling activators of murine microglia and human monocytes. Both miRNAs released from injured neurons into the extracellular space and miRNAs present in injured neurons were assessed within our study. Importantly, most of the miRNAs detected in supernatants and neurons by our screen approach harbored previously reported TLR7/8-activating sequence motifs. As a consequence, 83% of the miRNAs enriched in the neuronal media and therefore able to act as signaling molecules in principle, induced activation of human and/or murine TLR7/8.

In our current study, miR-128 family members, which are highly conserved and abundant in murine and human brain tissue [32], were deregulated in opposite directions. Although it is not clear whether deregulation of this miRNA species (and of all the other identified miRNAs) was directly involved (i.e., causative) in the apoptosis induced by staurosporine or was rather a consequence of apoptosis induction without being required for this process, the reduction of this miRNA species in neurons and their enrichment in the corresponding media may point to a specific release of these miRNAs. These findings are in line with the results of Pigati et al. who demonstrated that miRNAs yielded from epithelial cells and detected in circulating body fluids do not necessarily reflect the cellular miRNA content [33]. However, in case of the other miRNA candidates identified by our sequencing approach we did not observe such a deregulation. This discrepancy may be due to the limitations of our sequencing-based discovery approach and the fact that miRNAs can be enclosed in vesicular structures and/or protein complexes, which have been eluded by our nucleotide enrichment strategy. Interestingly, select miRNAs, such as miR-128, exclusively activated human TLR8, although their sequences do not match established TLR8-activating motifs [4]. In the context of lung cancer, Fabbri and colleagues reported on a systematic approach using NanoString technology, thereby identifying miRNAs present in exosomes from immortalized lung cancer cells as activators of TLR7/8 [30]. A further systematic study employing miRNA array technology identified miRNAs in murine blood plasma as a consequence of transient myocardial ischemia. In this work, the most potent miRNAs contained several uridines as a sequence feature and induced a cytokine response in macrophages and cardiomyocytes [31]. In accordance with our previous findings that miRNAs can be released from CNS cells, miRNAs enriched in supernatants of apoptotic neurons but also the ones that were less present in neurons harbored known TLR7/8 recognition motifs, as determined in our current report.

The concentrations of the synthetic miRNAs used in this study may be considered supraphysiologic and may not represent physiologic expression levels. However, to date it remains unresolved to which extent a given cell releases not only miRNA species, but also other RNA classes, which potentially act as signaling molecules. Likewise, the precise local concentrations of extracellularly functional miRNAs in the brain parenchyma at the site of injury/pathology in vivo are not known. Nevertheless, using various oligoribonucleotide concentrations and mutated control oligoribonucleotides we demonstrated dose- and time-dependent effects of select candidate miRNAs with respect to TNF release from microglia (Figure S3a–d), which stands in favor of a specific effect mediated by the respective miRNA. Additionally, it is conceivable that pathophysiological miRNA concentrations that trigger TLR activation could be achieved not only by one miRNA species, but probably by a mixture of different miRNAs acting in a combinatory manner (see Table 1 and Figure 2). Thus, the potential of miRNAs to synergistically activate and/or inhibit TLR-mediated immune responses should be addressed in future studies. In addition, recent findings suggest that cells can release miRNAs enclosed in exosomes, microvesicles, or in complex with proteins [9,30]. Here, we did not take account of possible molecular structures associated with the miRNA release to technically ensure the receipt of detectable amounts of miRNAs. As in our study cells were transfected in vitro, thereby likely facilitating the miRNAs’ cellular uptake and access to the endosomal compartment, the in vivo implications of our findings, in particular the molecular mechanism how extracellular miRNAs enter CNS cells (e.g., by phagocytosis), remain elusive. Further, we used synthetic miRNAs modified by phosphorothioate bonds in our activation assays, and although established as a stabilizing tool in miRNA research, we cannot exclude that these modifications have an impact on immune cell activation. Finally, the molecular mechanism underlying the neuronal injury triggered by extracellularly delivered miRNAs in the presence of microglia, remains unresolved, as this study focused on neuronal survival (i.e., loss of neurons in vitro) only. Overall, our data present a proof of principle demonstration of extracellularly delivered miRNAs acting as signaling molecules, and future work will be required to determine their pathophysiological concentrations and their mode of action dependent on dose and time in vivo.

As mentioned above, further miRNAs significantly reduced in apoptotic neurons, such as mmu-miR-410-5p, miR-124-5p, and miR-92a-1-5p, harbor previously described TLR7/8-activating sequence motifs suggesting a role as TLR activators, to be tested in future studies (see Table 1). As miR-340-3p, miR-132-5p, and let-7e-5p activated human TLR8, our findings may indicate a possible role of these miRNAs as TLR7/8 activators in human CNS diseases. Further, miR-128-1-5p and miR-128-2-5p are of special interest as (i) they are highly conserved and enriched in brain cells [32], (ii) their activation is restricted to human TLR8 (see Figure 2c), and (iii) their sequences contain no previously described TLR-activating motifs. Moreover, miR-128 was reported to be upregulated in brains of patients with Alzheimer’s disease (AD), in AD mouse models, as well as in cell culture models of Parkinson’s disease (PD). In addition, these miRNAs are discussed as potential biomarkers for neurodegenerative diseases such as AD [34,35,36,37]. In agreement with this, our recent work revealed that copy levels of the TLR7-activating miRNAs *let-7b* and *let-7e*—the latter also found to be enriched in media of apoptotic neurons in the current study—are elevated in the CSF of AD patients [10], implying a role for miRNAs as signaling molecules in neurodegenerative processes.

Our data point to a prominent role for miR-132-5p in TLR7/8 activation in the CNS. This miRNA is abundantly expressed in the brain and is known to exhibit regulatory functions in neurodevelopment, including neuronal differentiation, migration, and maturation [38]. Target genes of miR-132-5p include *Nurr1*, *PTEN*, and *FOXO3a* [38]. Dysregulation of miR-132-5p was linked to several neurological disorders including PD and psychiatric disorders. Upregulation of this miRNA was described in epilepsy, schizophrenia, PD, as well as in brains upon viral and parasite infection [38]. Additionally, increased levels of miR-132-5p in human B cells were associated with the onset of multiple sclerosis (MS) associated with a severe increase of proinflammatory factors such as TNF and lymphotoxin. The authors speculated about a mechanism of miR-132-5p action besides the established gene regulatory function [39]. In accordance with this, we hypothesize on a functional contribution of TLR signaling to MS and other neurodegenerative diseases mediated by miR-132-5p, acting in its extracellular state as a signaling molecule for TLR7/8.

We identified miR-340-3p as a highly potent activator of TLR7/8, capable of microglial activation and neuronal injury. Whereas the miR-340-5p form, which arises from the same pre-miRNA as miR-340-3p, was found to be negatively correlated with glioblastoma growth, and to act tumor-suppressive in lung cancer cells [40,41,42], only little is known about the function of miR-340-3p. Additionally, target genes of this miRNA are not clear yet. The potential of this miRNA to be released from cells and to activate TLR7/8 signaling may point to a role as a mediator of inflammatory processes that not necessarily have to be restricted to the CNS. In addition, miR-181a-1-3p detected in media from apoptotic neurons activated human TLR8. miR-181 family members have been previously described to act as tumor suppressors in human glioma and to trigger neuronal cell death in cerebral ischemia [43,44]. Interestingly, miR-181 is highly abundant in astrocytes and modulates neuroinflammation [18]. Thus, our data may indicate a role for the identified miRNA candidates as TLR signaling molecules for various CNS cell types.

## 5. Conclusions

In this report, miRNAs specifically released from apoptotic neurons were identified by small RNA sequencing. Select extracellularly delivered miRNAs including miR-132-5p and miR-340-3p activate TLR7/8 and induce the release of cytokines and chemokines from murine microglia and human monocytes. Further, these miRNAs mediate neuronal injury in co-cultures of microglia and neurons. The identified miRNAs activating TLR7/8 harbor novel sequence features crucial for receptor activation, as observed in miR-128 species, and require future systematic analyses. Further research will be required to investigate the probably highly complex role of miRNAs as signaling molecules in CNS injury, to establish clinical consequences of such injury including neurodegeneration triggered by miRNAs through TLRs in specific CNS disorders, and to identify means to prevent neurodegenerative consequences from such miRNAs and TLR signaling.

## Figures and Tables

**Table 1:** TLR7/8-activating microRNA (miRNA) candidates. Depicted are miRNA candidates enriched in the supernatant (log2FC ≥ ±1.0, *p* < 0.05) and miRNA candidates less present in neurons (log2FC > ±1.5, *p* < 0.05). miRNA family members are highlighted in grey. miRNAs contain hTLR8- and hTLR7/8-activating sequence motifs, as described by Forsbach et al. [4]. GU or AU content of individual miRNAs is given in %.

miRNA	Log2FC	*p* Value	Mature Sequence mirBASE 22.1	hTLR8	hTLR7/8	GU %	AU %
			Media				
mmu-miR-128-1-5p	2.9	0.0005	CGGGGCCGUAGCACUGUCUGA			57.1	33.3
mmu-miR-128-2-5p	2.6	0.0198	GGGGGCCGAUGCACUGUAAGAGA			56.5	39.1
mmu-miR-6240	1.8	0.0363	CCAAAGCAUCGCGAAGGCCCACGGG			34.6	30.8
mmu-miR-340-3p	1.4	0.0001	UCCGUCUCAGUUACUUUAUAGC	UUAUA	UCUC	54.5	59.1
mmu-miR-129-2-3p	1.4	0.0316	AAGCCCUUACCCCAAAAAGCAU			22.7	54.5
mmu-miR-7068-3p	1.3	0.0363	UCACCCUGGACUGACUCUCAG			42.9	42.9
mmu-miR-132-5p	1.1	0.0391	AACCGUGGCUUUCGAUUGUUAC		UUUC UUGU	59.1	54.5
mmu-miR-137-3p	1.1	0.0410	UUAUUGCUUAAGAAUACGCGUAG	UUAUU		56.5	65.2
mmu-miR-181a-1-3p	1.1	0.0000	ACCAUCGACCGUUGAUUGUACC	GUAC	UUGU	45.5	50.0
mmu-miR-181b-5p	1.0	0.0373	AACAUUCAUUGCUGUCGGUGGGUU			66.7	54.2
mmu-let-7d-5p	1.0	0.0000	AGAGGUAGUAGGUUGCAUAGUU			68.2	59.1
mmu-let-7e-5p	1.0	0.0013	UGAGGUAGGAGGUUGUAUAGUU	UAUA	UUGU	77.3	59.1
			Neurons				
mmu-miR-128-2-5p	−3.5	0.0000	GGGGGCCGAUGCACUGUAAGAGA			56.5	39.1
mmu-miR-128-1-5p	−2.8	0.0000	CGGGGCCGUAGCACUGUCUGA			57.1	33.3
mmu-miR-410-5p	−2.7	0.0072	AGGUUGUCUGUGAUGAGUUCG		UUGU	76.2	52.4
mmu-miR-409-5p	−2.7	0.0000	AGGUUACCCGAGCAACUUUGCAU			47.8	52.2
mmu-miR-124-5p	−2.6	0.0004	CGUGUUCACAGCGGACCUUGAU		UGUUC	54.5	45.5
mmu-miR-341-5p	−2.1	0.0051	CGGUCGGCCGAUCGCUCGGUC			57.1	23.8
mmu-miR-92a-1-5p	−1.9	0.0000	AGGUUGGGAUUUGUCGCAAUGCU		UUGU	69.6	52.2
mmu-miR-30f	−1.8	0.0051	GUAAACAUCCGACUGAAAGCUC			36.4	54.5

## Supplementary Materials

The following data are available online at https://www.mdpi.com/2073-4409/9/1/186/s1, Figure S1: Primary neuronal culture and small RNA enrichment strategy. Figure S2: Principal components analysis and identification of miRNAs differentially present in apoptotic neurons and corresponding media, Figure S3: miR-340-3p and miR-132-5p induce TNF release from microglia in a time- and dose-dependent manner, Figure S4: miR-340-3p and miR-132-5p induce cytokine and chemokine release from microglia, Figure S5: miR-340-3p and miR-132-5p do not affect neuronal survival in cell cultures of purified cortical neurons, Table S1: Deregulated miRNAs in neurons and media, Table S2: KEGG and Gene Ontology analyses.
