# Supplementary material for: Identification of CNS Injury-Related microRNAs as Novel Toll-Like Receptor 7/8 Signaling Activators by Small RNA Sequencing
Source: Cells. 2020 Jan 11;9(1):186. doi: 10.3390/cells9010186 (PMC7017345; doi:10.3390/cells9010186)
Supplement: Supplementary file 1 [file cells-09-00186-s001.pdf]

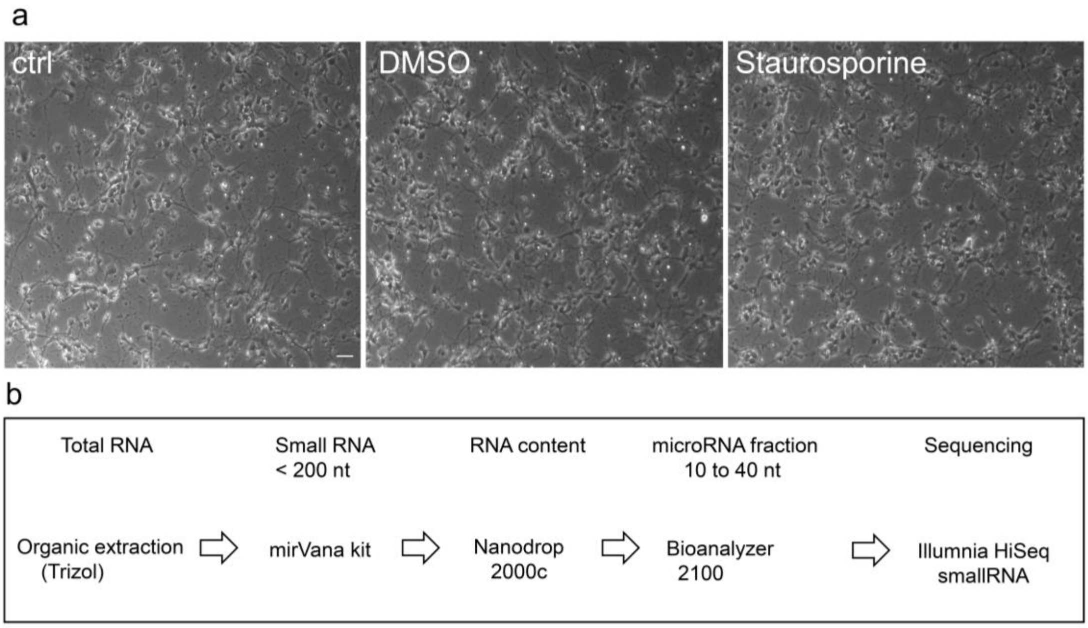

Figure S1. Primary neuronal culture and small RNA enrichment strategy. (**a**) Two million neurons isolated from C57BL/6 mice were incubated with 1 μM staurosporine or DMSO for 8 h. Neurons remained intact after the incubation with staurosporine. Control (ctrl) image shows neurons before incubation with DMSO or staurosporine. Scale bar represents 20 μm. (**b**) Organic total RNA extraction was followed by column-based small RNA enrichment. Photometric approaches were used to control quality and quantity of small RNA fractions. nt, nucleotides.

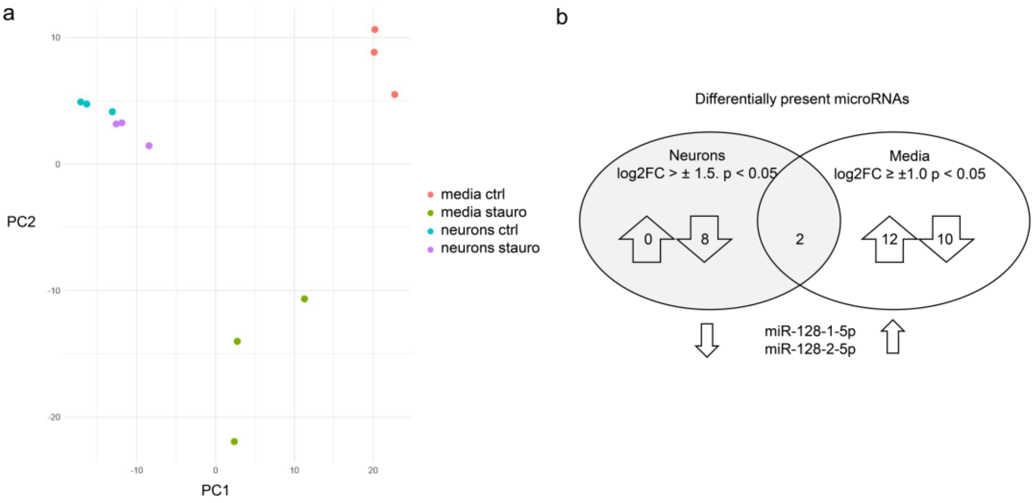

Figure S2. **Principal components analysis and identification of miRNAs differentially present in apoptotic neurons and corresponding media.** (**a**) PCA-Plot of analyzed samples showing the clustering of experimental groups into distinct populations. (**b**) Regulation of miR-128-1-5p and miR-128-2-5p in apoptotic neurons and corresponding media. Arrows indicate up- or downregulation. Numbers in arrows provide the quantity of different miRNAs, which are enriched or less present in apoptotic neurons and media.

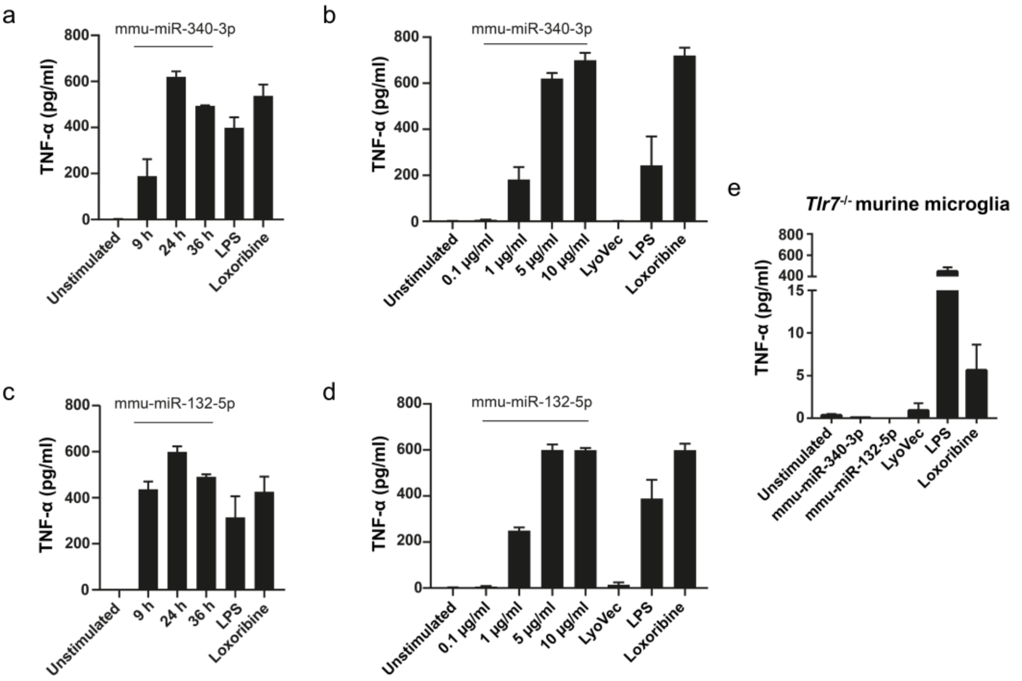

Figure S3. **miR-340-3p and miR-132-5p induce TNF release from microglia in a time- and dose-dependent manner.** Microglia from C57BL/6 mice were incubated with 5 µg/ml of mmu-miR-340-3p or mmu-miR-132-5p for various durations (**a,c**) or for 24 h with various dosages of mmu-miR-340-3p or mmu-miR-132-5p (**b,d**), as indicated. LPS (100 ng/ml) and Loxoribine (1 mM) were used as positive control, whereas LyoVec and unstimulated condition served as negative control. (**e**) Microglia from *Tlr7*$^{-/-}$ mice were incubated with 10 µg/ml of mmu-miR-340-3p or mmu-miR-132-5p for 24 h using the same control conditions as described above. TNF amounts in supernatants were determined by ELISA. Data of $n = 3$ are represented as mean +/- s.e.m.

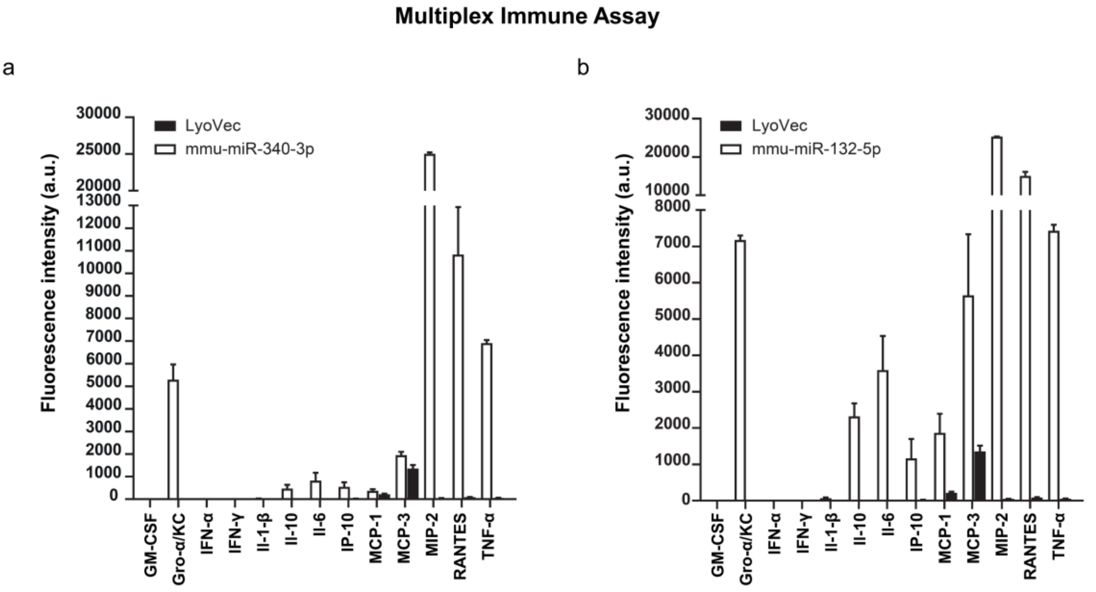

Figure S4. **miR-340-3p and miR-132-5p induce cytokine and chemokine release from microglia.** Microglia from C57BL/6 mice were transfected with 5 µg/ml of mmu-miR-340-3p (**a**) or mmu-miR-132-5p (**b**) for 24 h. Supernatants were analyzed by multiplex immune assay. LyoVec alone (black bar) was used as a negative control. Data are shown as arbitrary units (a.u.) of fluorescence intensity. Data of $n = 3$ are represented as mean +/- s.e.m.

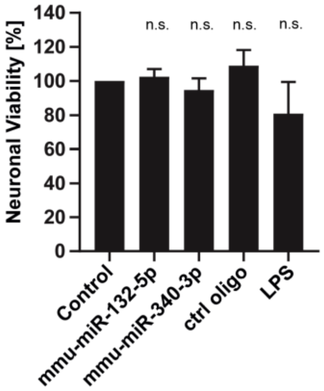

Figure S5. miR-340-3p and miR-132-5p do not affect survival of murine cortical neurons. Purified cortical neurons derived from C57BL/6 mice were incubated with 5 µg/ml of mmu-miR-340-3p, mmu-miR-132-5p, or control oligoribonucleotide (ctrl oligo) for 4 d. LPS (100 ng/ml) was used as a control for activation of potentially present microglia, while water served as volume control (control). Subsequently, cells were stained with NeuN antibody and DAPI. Relative neuronal viability was assessed by quantification of NeuN-positive cells. All treatment conditions compared to control were tested for significance by Student's *t*-Test. Data of $n = 3$ are represented as mean +/- s.e.m. n.s., not significant.
